# Regional Innovation in Arts Provision Spawned by COVID-19: “It Became a Lifeline for a Lot of People Who Are Stuck at Home”

**DOI:** 10.3389/fpubh.2022.753973

**Published:** 2022-02-17

**Authors:** Joanne Worsley, Josie Billington, Ekaterina Balabanova, Megan Watkins

**Affiliations:** ^1^Department of English, University of Liverpool, Liverpool, United Kingdom; ^2^Department of Communication and Media, University of Liverpool, Liverpool, United Kingdom; ^3^School of Health and Social Care, London South Bank University, London, United Kingdom

**Keywords:** COVID-19, public mental health, arts engagement, social isolation, cross-sector collaboration

## Abstract

Although the impact of the global COVID-19 pandemic on the arts and cultural sector due to the closure of galleries, museums, arts venues, and other cultural assets represents a significant health risk, new opportunities for arts and cultural engagement have arisen. Interviews with 24 representatives including service providers and creative practitioners from 15 arts and cultural organizations within the Liverpool City Region were conducted. The aim was to examine the impact of COVID-19 on arts and cultural provision and on organizations and people providing these services, as well as to understand the perceptions of service providers and practitioners of the effects on those whom arts and cultural organizations serve, including those who would usually access arts through formal healthcare routes (e.g., through collaboration with health partners). Interview data were analyzed using framework analysis. Four overarching themes were identified: Response: Closures, adaptations, and new directions; Challenges of online provision; Value of online provision; and the future of the arts. The arts and cultural sector has innovated rapidly, notably with accelerated digitalisation. Alternative provision has been “a lifeline” for vulnerable groups, such as those with mental health difficulties. Arts organizations have been most effective in reaching vulnerable, isolated and disadvantaged populations when they have worked in close collaboration with health and social care providers. The implementation of hybrid provision is an important move forward for the sector in light of our findings that alternative modes of provision are advantageous additions to service as usual. Given the increasing concerns about the mental health sequelae of the pandemic in the UK, arts and cultural engagement could play a pivotal role in the future recovery period.

## Introduction

In response to the global COVID-19 pandemic, in March 2020 the UK Government issued a nationwide lockdown to suppress virus transmission. Control measures such as closure of public spaces and restrictions on social contact profoundly affected day-to-day life. There is evidence to suggest that such measures resulted in adverse psychological consequences across general populations (1, 2). This poses important questions about how to prevent and mitigate those effects, particularly for vulnerable groups, such as those with pre-existing mental health difficulties. Early evidence suggests that vulnerable groups have been acutely affected during the pandemic (3, 4), and concerns have been raised in relation to the longer-term consequences of COVID-19 for these groups (5).

There is a growing body of evidence on the health benefits of engaging in the arts (6–8). Studies have consistently identified associations between arts participation (e.g., actively engaging in arts activities such as dancing) or cultural engagement (e.g., visiting museums, galleries or theaters) and the prevention, or reduced severity, of mental health conditions (9–11). Acknowledging this, even prior to the start of the global pandemic, Arts Council England (ACE) launched “Let's Create,” a 10-year strategy setting out objectives and investment principles for 2020–2030. It included a pledge to develop deeper partnerships with the Department of Health and Social Care and NHS England for the prescription of creative and cultural activities for mental health (12). Following the Creative Health report (13), the result of a 2-year inquiry led by the All-Party Parliamentary Group on Arts, Health and Wellbeing, a new national center for creativity and wellbeing was launched in March 2021, the National Center for Creative Health (NCCH). The NCCR is a strategic center that champions the role of creativity in health and wellbeing of individuals and communities. Through informing policy and promoting collaboration, the NCCH aims to make creativity integral to health and social care systems.

The impact of the pandemic on the arts and cultural sector due to the immediate closure of public spaces, galleries, museums, arts venues, and other cultural assets therefore represents a significant health risk. In-person arts and cultural engagement upon which a significant number of those from vulnerable populations relied for regular contact were ceased. Early evidence suggests that individuals with existing mental health conditions experienced poor mental health during the COVID-19 pandemic, and were unable to engage in activities that usually protected their mental health, such as accessing museums and theaters (14). The global pandemic has, however, provided new opportunities for arts and cultural engagement. In response to COVID-19 and the imposed restrictions, arts and cultural organizations began offering digital activities such as virtual tours, online groups (e.g., reading groups and virtual choirs), and streamed performances (e.g., plays and music concerts). The potential for arts and culture to support people's mental health and wellbeing is underlined by a recent study where hobbies including listening to music, reading, and engaging in arts activities were associated with reduced symptoms of depression and anxiety, and increases in life satisfaction during the COVID-19 pandemic (15). Arts engagement has also helped people to regulate their emotions during this time, demonstrating the value of the arts as coping tools during stressful situations (16).

The city of Liverpool has one of the richest concentrations of culture in the United Kingdom, with the largest clustering of museums and galleries outside of the capital. Culture and creativity, including (popular and classical, grassroots and elite) performing arts, music, theater, dance, museums, visual arts, events, and festivals, are central to the city's identity (17). Liverpool's Cultural Quarter, comprising Grade II listed buildings, the Walker Art Gallery and World Museum, was central to its former UNESCO World Heritage status^1^, and cultural capital is critical to the city region's economy, contributing c10%^2^. The Liverpool City Region (LCR) also has a history of harnessing arts for mental health care through partnerships between arts and health providers. One NHS Foundation Trust, a provider of mental health services across North West England, has a strong track record of working with non-NHS agencies to encourage social inclusion and enable people to enhance their health and wellbeing by enriching their life experience (18–21). Following the awarding of European Capital of Culture in 2008, the Trust in question nurtured a number of creative and cultural partnerships, which are now integral elements of the care offer. For example, in 2016, the Trust introduced the Life Rooms, a community service, to support the health and wellbeing of service users, carers, and local communities^3^. The Life Rooms is designed to support the prevention and population health agendas through a three-pillar model: social prescribing, community, and learning. Working in partnership with arts and cultural providers in the LCR (including a theater and a concert hall), the Life Rooms offers a range of creative courses, such as storytelling, creative writing, and music making. In the face of COVID-19, the Life Rooms service was restructured to provide a full suite of online and telephone support services, and a number of civic and community organizations deliver weekly arts courses as part of the Life Rooms online offer.

This paper reports the findings of a study that aimed to examine the impact of COVID-19 on arts and cultural provision in the LCR, on organizations and people providing these services, as well as to understand the perceptions of service providers and practitioners of the effects on those whom arts and cultural organizations serve, including those who would usually access arts through formal healthcare routes (e.g., through collaboration with health partners). Through delivering participatory programmes, arts providers and practitioners are well-placed to reflect on the impact of such provision on customary beneficiaries.

## Methods

### Ethical Approval

Ethical approval was received from the Central University Research Ethics Committee, University of Liverpool (reference number 7994). All methods were carried out in accordance with relevant university guidelines. The participants provided their written consent to participate in this study.

### Participants

Twenty-four representatives (22 females and 2 males) including service providers and creative practitioners from 15 arts and cultural organizations, including museums (*n* = 1), theaters (*n* = 3), galleries (*n* = 8), concert halls (*n* = 3), community and participatory arts organizations (*n* = 9) within the LCR participated. The majority of these organizations run participatory programmes for customary beneficiaries who are experiencing, or at risk of, mental health difficulties, often in collaboration with health and/or social care providers. Six of these fifteen organizations deliver provision in partnership with the Life Rooms, and a further two of these organizations have delivered provision with the Life Rooms in the past. Large institutions in the LCR (e.g., Liverpool Everyman & Playhouse, National Museums Liverpool, Liverpool Philharmonic, Tate Liverpool) were approached to participate, with additional arts organizations being identified and recruited through snowballing. As all arts and cultural organizations in the LCR were eligible to take part, our sample included both the long-established organizations listed above, and grassroots organizations.

### Data Collection

Interviews were conducted between September 2020 and May 2021 covering a period during which arts and cultural provision in LCR remained largely online. Following partial lockdown lifting in Summer 2020, restrictions were reintroduced in England during September 2020 (e.g., indoor and outdoor gatherings of six or more people were banned and there was a return to working from home). England entered the second national lockdown on 5th November 2020, which lasted for 4 weeks, after which a three-tier system of restrictions (stricter than those imposed in September 2020) was implemented. The third national lockdown commenced on 6th January 2021. Semi-structured qualitative telephone or video call interviews were conducted using a prepared topic guide that posed questions about the impact of the COVID-19 lockdown and restrictions on usual provision; the alternative measures put in place to try to continue service provision; the impact of restricted access and alternative provision on beneficiaries; and successes and challenges in this process (see Supplementary File 1). Interview duration ranged from 30 min to 2 h. With participant consent, the interviews were recorded and transcribed verbatim.

### Analysis

Interview data were analyzed through framework analysis, using the qualitative software, NVivo version 12. Framework analysis was selected as it allows for a combined deductive and inductive approach to data analysis, and has been endorsed for the management of data in health research (22). The procedure of framework analysis outlined by Gale et al. (22) was followed. Familiarization involved the process of transcription and repeated viewing of the transcripts. Line-by-line coding ensured that important aspects of the data were not overlooked. A subset of transcripts were read and coded independently by two researchers, who met frequently to discuss codes. A set of codes was agreed on and used to guide the coding of subsequent transcripts. Codes were grouped together into categories and defined to produce a working analytical framework. The framework was applied to all transcripts through indexing. Resultant themes and subthemes were renamed, refined and agreed by the whole team.

## Results

Four overarching themes were identified: Response: Closures, adaptations, and new directions; Challenges of online provision; Value of online provision; and the future of the arts. A number of subthemes were identified in relation to each overarching theme (see Table 1).

**Table 1:** Overarching themes and subthemes.

**Themes**	**Subthemes**
Response: Closures, adaptations, and new directions	*Closure of spaces: “People found this lifeline was taken away”*
	*Pivot to remote delivery*
	*Appropriate support and training for digital literacy*
	*Sense of responsibility: “It was the arts provision and social enterprises that picked up the pieces”*
	*Partnership working in the face of the pandemic: “It's really come to the fore in COVID”*
Challenges of online provision	*Barriers to online engagement*
	*Online safeguarding procedures*
	*Limitations of pre-existing technology and equipment*
	*Sense of loss*
Value of online provision: “A lifeline”	*Promotion of positive mental health and wellbeing*
	*Buffer against isolation and loneliness: Online provision “pulled everyone through it”*
	*Flexibility of online provision*
The future of the arts	*No “one size fits all” strategy: “It doesn't replace face-to-face”*
	*Moving toward a hybrid model of provision*

### Response: Closures, Adaptations, and New Directions

#### Closure of Spaces: “People Found This Lifeline Was Taken Away”

Theaters, galleries, museums, arts venues and other cultural assets are reported to be “a lifeline” for some people. Before the global pandemic, visiting creative spaces formed a vital part of people's social life and weekly routine. According to arts providers and practitioners, the immediate closure of public spaces necessitated by COVID-19 had negative consequences on beneficiaries' wellbeing:

*Not being able to come to (name of contemporary arts center) and access our garden and our gallery, and just a nice chat with some friendly staff members and volunteers, that might have had quite an impact on some people*… *It's like their safe place to come, essentially* (Participant 8, Contemporary arts center).

The implementation of the furlough scheme led to the suspension of usual services, which many vulnerable populations relied on for regular contact:

*Our Dance for Parkinson's programme stopped… Some of the people in that group told us that their conditions had even worsened through not coming in regularly to the class, which was really sad* (Participant 22, Dance organization).

Arts providers also highlighted that, as in-person cultural engagement and community arts engagement were entirely ceased, social isolation and loneliness increased:

*Within the Life Rooms, service users, for example, there were people who were veterans, who have suffered post-traumatic stress disorder and anxiety to such a level that they had not been able to leave their rooms or their accommodation… For them to be able to progress to come to the theater to engage and those people were so determined because they were participating often in events and courses that were really geared toward building self-esteem and confidence and managing anxiety… The moment that we close the doors of the theaters, their feedback was that they were terrified, that it was going to be imposed upon them to be isolated again and what that was going to do to their mental health* (Participant 6, Theater).

#### Pivot to Remote Delivery

In the face of the COVID-19 pandemic, the arts and cultural sector innovated rapidly, notably with accelerated digitalisation:

*Within 3 weeks we had turned around running shared reading groups on the likes of Zoom* (Participant 3, Shared reading organization).

Although many arts and cultural organizations were already considering and/or planning to implement a digital element to their offer, COVID-19 has been a catalyst in the growth and evolution of digital provision:

*We've all had a crash course in the feasibility and practicality of online delivery of arts… That probably wouldn't have happened with the speed or the sophistication… if that hadn't been driven by the necessity of a global pandemic* (Participant 24, Creative writer).

Innovations by arts organizations included creating new or adapting (e.g., shortening or restructuring) existing programmes:

*We have to structure activities a little bit more (on Zoom). For example, taking turns, playing musical games, and having more discussions* (Participant 12, Concert Hall).

Additional communication channels were set up to facilitate dialogue with beneficiaries in between live Zoom sessions:

*We have a Facebook group as well so we have quite a bit of dialogue back and forth through the week* (Participant 17, Photographer).

Although the digital arena has come to the fore, arts organizations also deployed offline approaches such as creativity and activity packs to reach those who do not have access to resources such as technology and internet connection:

*We've developed these Lifeline packs… which are packs of stories and poems posted out each week, semi prepped… with the odd question and prompt, so that if someone is shielding or not able to access any Zoom session or phone session… they could have a cup of tea and almost have a simulated experience of shared reading… Those packs were initially designed for people who were completely isolated and for people who might be also in prison not able to get out of their cell as well as people who might be in a kind of cell in their living room* (Participant 9, Shared reading organization).

#### Appropriate Training and Support for Digital Literacy

In light of these considerations, skilling up workforce and beneficiaries in technical knowledge and digital know-how was a key priority:

*We did get funding, we have been supporting people by giving them tablets with data on them and we're paying for monthly data for people… Some of them have never had a tablet before and we've had to do a lot of training and work with people to improve their digital skills* (Participant 15, Choir).

Although arts organizations received funding to purchase hardware and data for beneficiaries, it became evident that this approach needed to be supplemented with personalized, one-to-one support:

*We know that there are people that have come to our sessions at the Life Rooms in the past that are not yet able to access the online sessions, but the Life Rooms are doing everything they can to support those people and they're doing that in a very bespoke one-to-one way with individuals* (Participant 12, Concert Hall).

Some arts organizations also enlisted the help of beneficiaries' relatives to facilitate digital engagement:

*Staff members talking to parents and carers to help them support the person to engage with the activities… Our staff team are communicating literally on an individual level with every member every week in order to support, encourage, troubleshoot… help people. It's been very bespoke and very person centered* (Participant 8, Contemporary arts center).

Arts organizations also created instruction manuals or video guides to further support digital engagement. Due to the rapid pivot to online delivery, development of ways to encourage participant involvement is ongoing, and arts providers highlighted this as an area for further thought and consideration:

*The tools are there but the infrastructure to support engagement is still evolving and during COVID, it started to speed up a little bit but it needs to continue so that it can be inclusive* (Participant 2, Museum).

Artists, too, who were often delivering online for the first time, required digital training:

*Many of the artists we work with as part of that programme have never done a live sharing on Instagram before, so we've had to make resources such as “how to” guides* (Participant 19, Cultural and creative hub).

#### Sense of Responsibility: “It Was the Arts Provision and Social Enterprises That Picked up the Pieces”

Following the restrictions on social contact and reduced access to services, practitioners' sense of responsibility for beneficiaries' health and wellbeing was heightened. Many arts providers and practitioners “checked in” with their usual beneficiaries (hence the value of these providers' perception of the impact of COVID-19 on vulnerable people's mental health):

*We've done some extra stuff as well-like the little one-to-ones. One lady, I Facetime, an extra Facetime, once a week*… *We have the responsibility of delivering a weekly session creatively, but I feel like even regardless of the pandemic, there's a sort of element of responsibility for their health and wellbeing as well, and then obviously with the pandemic that just takes a whole new level* (Participant 17, Photographer).

*I was doing all of the official wellbeing calls… When I was phoning them I was making sure have you got food? Have you been to your GP? Have you got your prescription sorted?* (Participant 15, Choir).

Arts organizations also proactively reached vulnerable populations as usual support became unavailable. Some arts providers felt as though they were the social glue, stepping in when social services were not operating:

*It was the arts provision and the social enterprises that picked up the pieces where statutory services failed people completely… I spent a lot of time in those first couple of months sorting food out for people, making sure people had electricity* (Participant 15, Choir).

As it was common for arts providers to find themselves working in an unofficial way when social service provision was interrupted, it was noted that it would be beneficial to find a way of coordinating local activities and efforts:

*I think the difficulty is that if you haven't got anything that holds, almost like a register of all those bodies, together the local efforts, it's hard to know sometimes who can pull together all this goodwill and all this talent to make the most of it. It can feel sometimes like we're all running after the same thing, and we're all making up our own way of doing it* (Participant 3, Shared reading organization).

Despite all their efforts, some arts providers felt as though they were not going far enough to support beneficiaries; nevertheless, recipients were grateful for the alternative provision:

*I thought that maybe I wasn't doing enough, I needed to do something else to keep sustaining them. But surprisingly, they're so appreciative of really what I saw as not a major amount of (effort) on my behalf really. I'm not going out with them taking photographs… I'm not walking around galleries with them… They've been really very appreciative of all this level of contact, which I think probably goes to show potentially their level of isolation* (Participant 21, Photographer).

#### Partnership Working in the Face of the Pandemic: “It's Really Come to the Fore in COVID”

Arts providers were acutely aware of the need to work in partnership with other voluntary organizations as well as health and social care providers:

*We also then realized that we are going to have to work extremely well-with partner organizations… More than ever it's become vital that we collaborate well, particularly when every single organization has got its own infrastructure concerns* (Participant 3, Shared reading organization).

Arts organizations have been most effective in reaching vulnerable, isolated, and disadvantaged populations when they have worked in close collaboration with health and social care providers. Partnerships with health and social care services have made it possible for non-digital provision—in the form of creativity packs—to reach people in care homes, prisons, and inpatient mental health settings:

*We can email them (creativity packs) out to a range of partner organizations. So, particularly useful in care homes, mental health inpatient units where people are stuck there. But we know that if we can get something into the hands of somebody who works on these premises, they can print them and distribute them* (Participant 3, Shared reading organization).

Partnership working is also beneficial for health and social care staff as many have utilized arts and culture to support their own mental health and wellbeing during the COVID-19 pandemic:

*We've had lots of reports actually that staff use them (creativity packs) for their own wellbeing during coffee breaks. They have a read of them and just use that as an opportunity to unwind. It also gives staff and people who live in what we would call closed settings something extra to talk about* (Participant 3, Shared reading organization).

Arts organizations have also trained care staff *in situ* to deliver arts interventions whilst practitioners were unable to deliver provision on site:

*We're about to develop a new way of working, where artists will form little development triads with support workers from Belong… where perhaps they might be going to help somebody get up in the morning, or help somebody with their lunch, they would add on the option of doing an art activity for an hour* (Participant 8, Contemporary arts center).

Yet, some health and social care providers have come under increasing, unsustainable pressure. As arts provision in health and social care is often regarded as secondary to physical care, partners were required to prioritize frontline work, thereby losing “*all that extra stuff, the stuff that makes life a joy and not just a business of survival, I think that's a huge loss that we've seen”* (Participant 3, Shared reading organization). As health and social care institutions were directing their efforts to the frontline, partnerships with arts organizations appeared to be of low priority. This context made a potential solution to some of the barriers, such as training health and social care staff to deliver arts interventions, hard to implement. Voluntary sector organizations also had their own priorities and infrastructure concerns which impacted upon collaborations and delivery. Although there are clear benefits associated with partnership working in the face of the pandemic, the many challenges facing health, social care, and voluntary sector organizations have often hindered effective provision of arts interventions:

*We ran a physiotherapy for dementia programme, which obviously got canceled because the brain charity, who we partner with, had to focus on more frontline stuff so that stopped completely* (Participant 22, Dance organization).

The NHS Trust providing the Life Rooms (see Introduction) launched the Life Rooms Online in October 2020, enabling creative courses to be delivered via Zoom. Since October 2020, a number of arts organizations in the LCR have been delivering courses in partnership with the Life Rooms:

*The Life Rooms have developed this integrated digital provision, the digital Life Rooms, and have taken those creative writing sessions that I've been doing back over so now I run them for both, for the Everyman and Playhouse for the Life Rooms* (Participant 24, Creative writer).

Life Rooms learning facilitators contact service users about courses and provide bespoke, personalized support to their members:

*Because the sessions are hosted by the Life Rooms, it was the Life Rooms that made that initial contact and asked people how they feel about joining Zoom* (Participant 12, Concert Hall).

Although creative courses delivered as part of the Life Rooms Online learning offer are delivered by a creative practitioner, a Life Rooms learning facilitator also supports each session. Arts practitioners delivering online provision, both as part of the Life Rooms provision and in partnership with other health and care providers (e.g., hospitals), highlighted the importance of teamwork as having a health or social care staff member present during online sessions facilitated effective delivery:

*We had some successful sessions, and we had some sessions that were just not working. I noticed a pattern that when the staff at the (name of in-patient children's mental health unit) was not present on the online session, it was just not the same. And so that really indicated for me the crucial need for online delivery when it comes to addressing more personal sessions, that you really need a member of staff on board*… *What's really meaningful for me, overall, is this teamwork I have now with the members of staff… I think we can really learn from each other, the Life Rooms facilitators, the management at the Philharmonic, and myself. I think it's been a real benefit for us to work together as a team around the service users* (Participant 18, Musician).

### Challenges of Online Provision

#### Barriers to Online Engagement: “It Is Genuinely Like Rebuilding a Movement”

Following the pivot to digital platforms, many populations remained out of reach due to limited technological literacy or access to digital resources:

*Not everyone has a computer, not everyone has Wi-Fi, they may have data on their phone, but it's limited in relation to being able to attend an hour or 45-min class that might take up their whole data for the month* (Participant 10, Dance organization).

*There were people who we were working with at the Life Rooms who definitely valued the sessions… who suffered from digital poverty. They (“people who had been regular attendees of the in-situ sessions”) did not necessarily have smartphones or all the IT equipment necessary to attend the digital sessions*. (Participant 24, Creative writer).

In addition to lack of access to hardware and/or data, other barriers to engagement highlighted included lack of digital know-how, “*bad*” or “*fluctuating internet connections*” (Participant 11, Theater), and “*Zoom fatigue or screen fatigue*” (Participant 19, Cultural and creative hub). Examples of out of reach populations included refugees, asylum seekers and street-based sex workers. Work with people seeking asylum has “*ground to a halt*” due to a combination of “*reduced likelihood that people can access internet or equipment… services being shut and the added difficulty… if people don't have confidence in English language skills”* (Participant 3, Shared reading organization). Both younger and older populations were also highlighted as groups that were hard to reach for different reasons. For young people, digital fatigue was commonly cited as a barrier to engagement:

*Our younger audiences… have probably struggled the most because although they may be more likely to have the physical online tools and know how to engage, there's been a barrier in terms of… just struggling with what's going on in general, and… if they're young people that are also in education at the time their entire education had move online as well. So (as) they were already spending all day online being taught, the thought of them doing something on a weekend or of an evening online probably was the last thing that they had in mind* (Participant 14, Photography gallery).

For older populations, attitudinal as well as technological barriers were commonly reported:

*Particularly for people over the age of 65… it's not just about having the right equipment or having the internet. I think it's also about confidence, it's about feeling like technology can be unsafe, because it's quite unfamiliar* (Participant 12, Concert Hall).

However, although older people were initially reluctant to engage via online means, attitudes changed over time as many customary beneficiaries realized that digital provision was not a temporary measure:

*As everybody realizes this isn't going to go away as quickly as people thought at the beginning of the pandemic… I think those people with those intellectual or attitudinal barriers to going digital, slowly melting and accepting that they might need to go online in order to continue to connect with those around them* (Participant 11, Theater).

Lastly, digital exclusion affected not only access to arts provision but communication of information about arts provision. The impact was two-way as it was impossible to gauge how many usual beneficiaries accessed certain online services such as Facebook live streaming. One arts provider suggested that reconnecting with beneficiaries would be a challenge:

*You could have the most incredible, easy to use, life changing therapeutic intervention, but if you can't actually get it to people, it's almost as much use as if it doesn't exist*… *Getting to those people who are suddenly completely off our radar. Where are they? How is life for them? Why can't they join a call? … I think the slightly sobering thought has been we've put so much work over the years into reaching people who are the very hardest to get in touch with… We've put such a lot of effort in over the years to try and get through barriers with people, and then to be presented with not only losing an awful lot of our readers through a situation that none of us could have foreseen, but not being sure how to get back in touch with them it is genuinely like rebuilding a movement* (Participant 3, Shared reading organization).

#### Online Safeguarding Procedures: “A Minefield”

Safeguarding in digital spaces has required a lot of attention and thought, especially for sessions delivered for vulnerable populations in collaboration with healthcare providers. Arts organizations were acutely aware of this urgent need:

*Safeguarding is obviously one of the most pivotal and primary concerns, and anyone that works with young people in organizations like this, there's another world of training and procedures and risk involved with this* (Participant 20, Music organization).

Many organizations highlighted that there were two members of staff present during each Zoom session, which differed from usual provision. For example, courses delivered via the Life Rooms' online learning platform were usually facilitated by both a creative practitioner and supported by a Life Rooms facilitator “*taking people out of the chat if people want to talk or there's a wellbeing issue”* (Participant 24, Creative writer). Most organizations devised a formal set of guidelines or ground rules, which were introduced to participants at the start of each Zoom session:

*They (the Life Rooms) have quite set rules for it and they go up on a slide at the beginning of the session. Most of them are what you might expect so please don't come if you're drinking or under the influence of drugs, don't smoke or vape on the camera, be respectful. Make sure you're the only person in the room and that people can't be seen because obviously there's safeguarding issues there* (Participant 24, Creative writer).

Procedures in terms of copyright law were also highlighted as being more critical when operating in the digital arena:

*We've also… very quickly realized the impact of copyright law when we can't use an educational license which we usually operate under which allows us to replicate text to certain percentage, as long as we're only sharing them within a small group* (Participant 3, Shared reading organization).

*If we're using an artist's work, we have to get permission to use it. But in an online context, it's even more important that we get that copyright* (Participant 4, Art gallery).

#### Limitations of Pre-existing Technology and Equipment

Although many arts organizations pivoted to platforms such as Zoom to deliver arts activities, this software was not created for this purpose. As Zoom has technological and capacity limitations, using this software for certain artistic pursuits proved challenging:

*You can't sync the audio, often the audio cuts out, you've got to familiarize yourself with the settings on the actual Zoom to make sure that doesn't happen as much but it's still going to happen just because of the very nature of the work. You can't remove the problems of time and internet connections* (Participant 20, Music organization).

*If you want to keep a hold of your lessons you have to then transfer them to Dropbox or download to your own laptop* (Participant 22, Dance organization).

Following the model of the wellness industry, one arts organization invested in new software, Namastream, with a media library for video storage enabling beneficiaries to revisit past sessions:

*Within that portal, they can re-watch all of the classes that they paid for and it integrates with Zoom. So, when your live event takes place, that just means that it just goes live on Zoom* (Participant 22, Dance organization).

As indicated above, arts forms that were more difficult to reproduce online include music making and choir singing, and the impact upon the experience of such activities in the online milieu has resulted in fewer customary beneficiaries engaging:

*Music improvisation online is really complicated to organize because of sound issues and delay… The technology is not quite there yet to have eight young members together trying to have a jam session* (Participant 18, Musician).

*Rather than it being a choir, hearing each other's voices, singing together, singing in harmony, what we effectively have is our choir director singing, and we all have to be on mute because of the feedback issues that Zoom has, so… We've got 60 members in total and there's only ever about 25 that will take part in Zoom, and we are finding that across all of our choirs in the different cities. And that's not because of tech access because as I said, we've made sure everybody's got what they need. It's just that it's a real different experience and it doesn't suit everybody* (Participant 15, Choir).

Arts providers and practitioners also highlighted the pressing need for better equipment to enable high-quality delivery:

*We need much more reliable equipment to be working from with much higher processing speed and much better internet connectivity within it, especially when you're working with vulnerable people* (Participant 6, Theater).

*I was asked to provide recorded concerts. It was hard because I'm not used to doing this with my phone and very poor recording equipment* (Participant 18, Musician).

#### Sense of Loss

Arts providers and practitioners highlighted a sense of loss in respect of the wider experiences surrounding arts and culture. For example, arts providers struggled to recreate the important social aspects of in-person provision:

*Our rehearsal in real life is normally three hours… There's only an hour and a half of actual singing, the rest is hanging out together and eating the meal. So that sense of spending time with people has gone. I think that's really vital for mental health* (Participant 15, Choir).

*I think we are getting better at creating (social time) within interventions and workshops and performances but that's been the biggest hurdle I think to overcome, the cup of tea time, the let's have a biscuit together and find out how everybody is, because time feels a little bit more under pressure online* (Participant 11, Theater).

With contact time often shorter in online spaces, ancillary aspects of the social encounter which enriched face-to-face provision were also excluded:

*I'm not in the rehearsal room a lot of the time. I'm outside the rehearsal room sat with one person at a time because people come in, and they want to talk to you, they want to ask for help for something, so you can pull them out of the rehearsal, and have that one-to-one time, whereas you can't do that in the middle of a Zoom so there's a lot of connection that's lost* (Participant 15, Choir).

*A workshop might take up 2–3 h previously. Now a workshop will probably last somewhere between an hour and 15 min… So, you are losing a little bit of that wraparound support* (Participant 11, Theater).

Arts providers and practitioners also emphasized a sense of loss in respect of the personal connection and direct intimacy of meeting, which made identifying signs of distress, or silently providing comfort or support when the right language was hard to find or say, problematic:

*It's also a lot harder to catch the things that are happening to people in an ad hoc way, in a more organic way … Sometimes when you're together doing something people might not have said, I'm really struggling right now but you can see it in them … On Zoom, you lose that tactile nature of being able to offer help* (Participant 15, Choir)

*I always remember… a group were meeting together (in person) in one of the mental health inpatient wards in Liverpool… and there was a moment in the session where someone became moved, and started to quietly cry for a time… The group member who was sitting next to them did this kind of movement, when people put their hand up in a friendly fish shape to touch each other's fists, knock each other's hands together as a non-verbal, physical way of saying, “I get you, it's tough I'm here.” And so words are very, very difficult whether that's because someone is very emotionally fragile or upset or it's due to a cognitive impairment. And that's when those non-verbal moments which are so reliant on the physical are so precious… How can you replace something like that a hand on the shoulder or a physical leaning in but that's not to say that people we still can't provide meaningful ways to connect* (Participant 9, Shared reading organization).

The experience of the art activity as a (literal) “journey” which changed the participant was also missed in the move to digital delivery:

*That sense of traveling to somewhere, to a workshop, something happens in that workshop… something transformative… and the sense of then walking home and moving away from that. You lose all of that online* (Participant 16, Creative arts organization supporting writing).

### Value of Online Provision: “A Lifeline”

#### Promotion of Positive Mental Health and Wellbeing

According to arts providers and practitioners, online provision helped beneficiaries maintain positive mental health and wellbeing:

*In a couple of pockets in the UK, we've started shared reading over the phone for people who are temporarily housed in hotels. We've got some really interesting feedback from that actually that the difference between the wellbeing of residents in hotels that hadn't managed to implement any arts-based therapeutic groups and the hotels where they had. So the likes of The Reader doing online groups of people to access or reading with people over the phone, there has been a huge difference in the wellbeing of those participants* (Participant 3, Shared reading organization).

This seemed apparent particularly at the onset of the pandemic; one partner reported, there appeared to be “*an enormous number of people that fall in between the services that exist. The offer that we have has really helped people to maintain their mental health and wellbeing or to prevent them from going into crisis… Someone said it was a miracle. Called it a lifeline”* (Participant 6, Theater). One possible explanation for this is that engaging in arts activities has proved a valuable way to resist some of the pessimistic emotions spawned by COVID-19: “*They were using photography as a way to document what was going on for them, and it became quite a cathartic process for quite a lot of them (who) do talk around effects on wellbeing*' and see this type of project “*as a therapeutic process to counteract those negative feelings of the lockdown experience*” (Participant 14, Photography gallery). The acquisition of new skills and sense of accomplishment through skill-building was also highlighted by arts providers and practitioners in relation to both promoting recovery from mental ill health and in creating the digital know-how and confidence to access other forms of support or opportunities:

*The fact that we can all do this online is, in my opinion, triggering much more interest for the service users. I feel people are much more engaged than with my normal courses in researching for themselves and this is absolutely crucial for mental health recovery* (Participant 18, Musician).

*Some of them have never had a tablet before and we've had to do a lot of training and work with people to improve their digital skills, which then opens up everything else… Because once you've used it, and got comfortable with it in a supported environment, you're then able to use that in other places* (Participant 15, Choir).

Finally, engaging in weekly online provision was reported to have provided beneficiaries with a sense of routine, which is an important consideration for those experiencing or at risk of mental health issues:

*People really appreciate the regular activity (of) having something each week, and something to look forward to as well* (Participant 12, Concert Hall).

#### Buffer Against Isolation and Loneliness: Online Provision “Pulled Everybody Through It”

Arts providers and practitioners thought that alternative provision had been vital for those who were vulnerable, isolated, or disadvantaged, providing “*a lifeline for a lot of people who are stuck at home”* (Participant 16, Creative arts organization supporting writing):

*This person said, “the Reader leader on the phone was patient with me. It was a highlight of my week getting a call… Working with her during this time has been a salvation”* (Participant 9, Shared reading organization).

The extent of isolation felt by some beneficiaries illustrated the importance of on-going provision in people's social life:

*One of the women said to me… that she realized that the week before that she hadn't spoken to anybody for a whole week… She found herself talking to the wheelie bin* (Participant 21, Photographer).

*One lady said that she'd gone for a flu jab and the nurse held her arm and she said that's the first human touch she's had for months and she said “I really enjoyed it, she just held my arm.” That's when you realize how isolated the group have been individually. So I genuinely think it's actually pulled everybody through it in a way* (Participant 17, Photographer).

The interviews suggested that online arts provision provided a buffer against loneliness, especially for those who lived alone with limited opportunities to interact with others, by reducing isolation and providing meaningful activity:

*Although of course it's a good thing for (otherwise homeless) people to be housed and to be protected from the virus, it is a huge change for people, and particularly when we're under very strict lockdown guidelines or restrictions, just suddenly (to) sit in a hotel room 24 h a day it comes with its own complications* (Participant 3, Shared reading organization).

*Obviously, it's different than being in real time in real space but it's still a connection* (Participant 11, Theater).

Customary beneficiaries were explicitly appreciative of the opportunity to interact with others whilst participating in arts activities via digital means:

*Different service users began to really support each other… people were writing about and processing the emotions that were coming up over the course of the pandemic year, whether that was grief or anger at the government or feelings of loneliness… Through talking about each other's work, there was often quite a lot of concern and discussion about shared feelings and shared affects and shared experiences. I think a lot of people value that perhaps more than the actual nature of the writing exercises themselves* (Participant 24, Creative writer).

The importance of the group for providing a support network and sense of community was also highlighted by arts providers and practitioners:

*What happens now is that when somebody is having a crisis, it's developed from automatically coming to me, to now actually saying in the (Whatsapp) group, “I need help,” and the group members are able to then support each other a lot more* (Participant 15, Choir).

*You're offering each other ideas and you're sharing opinions about literature and about writing but there's also forms of care and support that emerge quite organically through that* (Participant 24, Creative writer).

Thus, although people were not together physically, there was still a sense of togetherness:

*Even though we're not in the same room, I'm going back to this idea of this incredible intimacy we are having with each other on a weekly basis because we're managing to really share this. We're all in isolation and we're managing to really come together in a truly meaningful way* (Participant 18, Musician)

*I don't think we would have ever chosen to have worked in this way, because so much does hinge on bringing people together but what we have learned from this is that that sense of togetherness doesn't necessarily rely on physicality* (Participant 3, Shared reading organization).

#### Flexibility of Online Provision

One advantage of online provision was the flexibility it offered to arts organizations, allowing them to reach new audiences, who had previously been unable to attend activities in person due to health, social anxiety, location or caring responsibilities. “The Reader at Home,” for example, made it possible for first-time beneficiaries to experience shared reading online:

*We have seen increases in certain pockets. For example, I have quite a bit of feedback from people saying that a mobility issue or perhaps even living with anxiety or lack of confidence would have previously inhibited them from coming along to a group in a public setting* (Participant 3, Shared reading organization).

As geography was no longer a barrier, a far greater (even global) reach was now possible than local *in-situ* provision previously allowed:

*It's definitely helped us develop in reaching out to audiences, not just in Liverpool, not just nationally, but internationally as well. Because once your work is online, it's then accessible to the whole world and you can connect in so many different ways* (Participant 23, Dance organization).

This resulted in a greater diversity of beneficiaries, and also enabled arts organizations to draw on a wider pool of resources, such as practitioners living in different cities as well as countries. This, in turn, enhanced their offer and providers described a desire to continue with online initiatives moving forward:

*What it has done is open us up to not needing to use local teachers… It's allowed us to have guest teachers from all over the world* (Participant 22, Dance organization).

*I think how the festival turned out online… is just a huge, huge success bringing national and international writers to a virtual stage in Liverpool was huge and the audience numbers, people obviously want that conversation and that debate…Will change the way that we work forever* (Participant 16, Creative arts organization supporting writing).

Arts organizations also reached more people within health and social care contexts, such as in-patient wards and prison settings, through their digital offer, as well as increasing the frequency of their provision:

*If we're actually making digital work that's good quality, it enables us to really expand the underrepresented audiences that we'd really like to reach. For example, if we get our arts council recovery fund… one of our plans is to make our Christmas show, which will be live at the theater but also digital. (Name of Children's NHS Foundation Trust) is a partner of ours and our plan would be to broadcast it through (Name of Children's NHS Foundation Trust), which actually over Christmas could potentially reach 1,000 children that are in hospital. With (NHS Foundation Trust), we've had a conversation about how we might be able to do that with them as well-because of their reach to secure settings and in settings where people just can't access theater* (Participant 6, Theater).

*We used to have wards from (name of NHS Foundation Trust) local division that would come to Philharmonic hall each month for that activity and now we're going to be doing Zoom sessions for those wards. So it's instead of them coming each month, we're going to be hosting fortnightly sessions, actually for more wards … so we'll be reaching more people* (Participant 12, Concert Hall).

### The Future of the Arts

#### No “One Size Fits All” Strategy: “It Doesn't Replace Face-to-Face”

Some beneficiaries preferred face-to-face provision, whilst others preferred the digital alternative; a “one size fits all” strategy may not be appropriate following the pandemic:

*There is a group of people who are absolutely desperate to get back in the studio, and a group of people who love the online provision and have found it fits really well into their lifestyle* (Participant 22, Dance organization).

Online activities enabled beneficiaries to participate from their home environment where they felt comfortable and not restrained for physical or personal reasons (see Flexibility of Online Provision). For others, although they were happy to participate digitally whilst unable to meet in person, they were looking forward to reconnecting in physical spaces “*as soon as we can*” (Participant 17, Photographer):

*It is working because they think at some point it will be at an end. Everybody says, “…when we can meet as a group, we are all going to be in Birkenhead park again”… they're happy to do this as long as they know that it's sustaining them because they think that in May we're going to be sitting back in a park* (Participant 21, Photographer).

Face-to-face provision and sharing an experience together in a physical space was regarded as irreplaceable and could not be supplanted satisfactorily by digital engagement:

*Bluecoat's very place based. It's about an experience, and that is very, very important* (Participant 8, Contemporary arts center).

*Nothing will replace real life. That's something I'm a bit scared about in a way, that humanity won't be lost with all these successes of delivering online. There are so many positives, but I hope it won't impact on all this crucial human experience that we all need* (Participant 18, Musician).

While there needs to be acceptance that there cannot be exactly the same provision online as in-person, interestingly, volunteers and participants have found that when a group activity has been set up online from the outset, it has been easier to navigate than a pre-existing group which has pivoted online:

*It's been actually more challenging for groups who've been used to meet[ing] in person [to] transition on to new platforms, compared to completely new groups that have been set up during the pandemic* (Participant 9, Shared reading organization).

#### Moving Toward a Hybrid Model of Provision

There is now a greater appreciation of the value of arts and creativity for health, and the pandemic has been a catalyst in driving this forward:

*In times of crisis, creativity is at the forefront of supporting us and to make us feel better* (Participant 16, Creative arts organization supporting writing).

*People need the arts. It's not a luxury or a privilege. It's a necessity to function in life*… *Especially when people don't have an outlet, they can't go out, their normal day-to-day lives have completely changed, our lifestyle has changed and so it was found that dance was essential for their wellbeing* (Participant 23, Dance organization).

Alternative modes of provision are advantageous additions to service as usual, and many arts organizations shared their intent to retain digital provision for the future, especially as there are advantages offered by technology:

*There are good reasons to do digital provision anyway as people have issues with mobility and other sorts of vulnerability that mean they can't necessarily attend in situ sessions. But I think going forward, I think the new will be to have both in situ and digital sessions because they complement each other, and I'm not sure necessarily that it'll be the same people using both. So in that respect, I'd say it has maybe transformed delivery* (Participant 24, Creative writer).

The Life Rooms online learning offer provided by an NHS Trust will also be available following the pandemic, enabling a number of creative and cultural organizations to continue to deliver their creative offer via this platform:

*What we realized is that going forward, both organizations [arts organization and NHS Trust] have made a commitment [to working] in this way. We need hybrid programmes. We need live and digital* (Participant 6, Theater).

One arts provider suggested that beneficiaries will have a choice in the future:

*We don't plan to stop the online programme just because the world opens up, I think we will now always have an online programme, but then there'll also be the option to come into the studio. What we're hoping to do at some stage is integrate the two so that there'll be cameras in the studio so that you can attend a live class that's happening in the studio from your home* (Participant 22, Dance organization).

As organizations planned for a rebalanced provision with greater online capacity, many arts providers were acutely aware of the need to evaluate “what is working” for the populations they serve:

*For January to March, we had a set of classes in place. We assessed how they did, who came to them? Which ones were really successful, which ones weren't? And for April, we've slightly adjusted the programme to fit our findings* (Participant 22, Dance organization).

*We're carrying out a COVID special evaluation in October, getting some feedback from our volunteers and group members as much as we can get in touch with them about what's worked well, what hasn't, what they miss, what they want*…*I think the real review actually is once you've got a core temporary model* (Participant 3, Shared reading organization).

Planned changes in delivery models and infrastructure were also being implemented in the context of budget cuts:

*We're going to be much more digitally savvy in the future and we will have to be adaptable and flexible in that… All Tate galleries had a 50% budget cut right across the board, so we have less money. So it's not like we've got more money to put into digital, we don't* (Participant 4, Art gallery).

## Discussion

This study set out to examine the impact of COVID-19 on arts and cultural provision in the LCR, on organizations and people providing these services, as well as to understand the perceptions of service providers and practitioners of the effects on the mental health of those whom arts and cultural organizations serve. Our key findings fall into three main categories: innovation and collaboration, challenges of online provision, and positive impacts.

The arts and cultural sector has innovated rapidly, notably through accelerated digitalisation. COVID-19 has been a catalyst in the growth and evolution of digital provision, and arts and cultural organizations in the LCR have responded with ingenuity, creating new bespoke services or adapting existing programmes to reach their usual as well as new audiences. Cross-sector collaborations between arts, health and social care services have been important in reaching vulnerable, isolated and disadvantaged populations, especially those who are experiencing or at risk of mental health difficulties.

Arts organizations have, however, encountered challenges. Certain populations have remained out of reach, such as the elderly, asylum seekers, and street-based sex workers, due to unfamiliarity with devices or lack of access to technology. The impact of digital exclusion has been two-way as some usual beneficiaries may not be aware of the alternative provision and arts providers cannot be sure how many customary beneficiaries are accessing certain forms of online provision (e.g., Facebook Live Streaming). As contact time is shorter online and excludes ancillary aspects of the face-to-face social encounter, service providers and practitioners reported encountering difficulties identifying signs of distress and providing “wraparound” support.

Previous research has shown that participation in arts and cultural activity can promote mental health and wellbeing (7, 15), and our findings based on the views of arts providers and practitioners support this evidence by illustrating the role of online arts provision in enhancing wellbeing and reducing feelings of isolation. Engaging in arts and cultural activities online was reported to have enabled beneficiaries to escape from negative emotions aroused by the pandemic, whilst also developing a sense of accomplishment through skill-building.

These findings will have implications for the sector, locally and nationally. The restrictions imposed in response to COVID-19 have made evident the importance of arts and culture for people's mental health and wellbeing. This recognition provides an important opportunity to capitalize on the role of arts and culture in prevention and recovery, contributing to solutions for health systems, which are currently under increasing, unsustainable pressure (23). In line with the 10-year strategy proposed by ACE (12), cross-sector working between arts and health sectors should drive future innovation. The Life Rooms, as an established and successful model of integrating arts and culture within NHS provision, offer a representative model of the adaptations and opportunities for arts-in-health delivery, driven by COVID-19 restrictions. The Life Rooms approach illustrates how, led by NHS Trust innovation, arts and cultural organizations can play a role in supporting health care (24), and might offer therefore an exemplar model for the ways in which arts organizations can work creatively with an NHS organization to support local communities. The Life Rooms innovation could provide a blueprint for similar collaborations in other regions of the UK. As arts and health sectors across the UK struggle with the on-going impact of the COVID-19 pandemic, greater investment in cross-sector collaborations is essential. In line with this, as there are currently many freelance creative practitioners out of work and a burgeoning mental health problem in the UK (25), it could be beneficial for these sectors to align more closely to support people's recovery in the aftermath of the pandemic.

Arts organizations often operate in silos, with local initiatives and efforts invisible to key stakeholders. Consistent with the LCR's Economic Recovery Plan (26) and the LCR Culture and Creativity 30 Year Strategy (27), our findings illustrate the need to coordinate local initiatives in the LCR, as coherence around a city region approach would be fruitful. During the initial lockdown period, many arts organizations operated in an unofficial way as social care services were temporarily interrupted. Arts providers highlighted the need for a “backbone organization” or “register” of providers to coordinate services and share best practice. A database of best practice and innovation in arts provision, including different modes of activity, for use by regional stakeholders including arts providers, health or link workers, and beneficiaries would be useful if social prescribing or “arts on prescription” is scaled-up to support population health and wellbeing in the aftermath of the pandemic. A database of best practice and innovation would be useful for arts organizations to link and collaborate with one another, but also for health or link workers to aid the prescription of arts activities. Thus, coordination of this kind would prove to be valuable in shaping arts on referral projects.

With regard to mode of delivery, our findings suggest that a “one size fits all” strategy may not be appropriate following the pandemic as some beneficiaries reportedly prefer online provision whilst others are keen to re-engage in-person. In light of this, digital provision is no longer considered to be a periphery way of accessing the arts, and many organizations in the LCR are currently planning for a rebalanced provision showing full commitment to a hybrid delivery model. As arts engagement is a valuable experience, giving people the option to access live provision either face-to-face or via online means would be beneficial. Resources should be directed toward such innovation. It should also be noted that arts organizations are currently delivering their provision via software designed for businesses (e.g., Zoom); however, our findings clearly illustrate limitations of using pre-existing technology to deliver arts interventions. If online provision is to continue and form a substantive part of the delivery model in the long-term, advancements in technology are urgently required.

These findings should, however, be considered in light of a number of limitations. First, our sample was composed of service providers and creative practitioners from the Liverpool City Region, a distinctive region with a history of harnessing arts for mental health care. Thus, our findings may have differed if we had included a more geographically diverse sample. Learning from this work, however, could be transferrable. Interviews were conducted between September 2020 and May 2021, and therefore do not capture experiences outside of this timeframe. Our data covers a range of civic and community arts organizations. While this provides a breadth of different experiences, it may limit the specificity of the findings. Those employed in civic institutions may have been impacted in different ways to those working in community arts organizations. Finally, our understanding of the impact of alternative provision on beneficiaries' wellbeing has been outlined by service providers and creative practitioners, rather than beneficiaries themselves. Future research should explore the perspectives of beneficiaries who participate in weekly online courses, such as service users who access arts provision via the Life Rooms online learning platform.

To conclude, COVID-19 has been a catalyst in the growth and evolution of digital provision, and the response in the LCR has been highly creative and collaborative. Some arts organizations have operated in an unofficial way to support beneficiaries as social care provision was interrupted in the face of the COVID-19 pandemic. As arts and cultural engagement could play a pivotal role in the future recovery period, our findings demonstrate the need for comprehensive financial and infrastructural support for provision that will be as vital for people's recovery in the aftermath of the pandemic as it has been “a lifeline” during the COVID-19 crisis.

## Data Availability Statement

The raw data supporting the conclusions of this article will be made available by the authors on reasonable request.

## Ethics Statement

The studies involving human participants were reviewed and approved by the Central University Research Ethics Committee, University of Liverpool (reference number 7994). The patients/participants provided their written informed consent to participate in this study.

## Author Contributions

JB and EB conceived the study. JW collected, analyzed the qualitative data, and wrote the first draft of the manuscript. MW coded a subset of transcripts. JB, EB, and MW read, commented on, and revised the manuscript providing important intellectual input. All authors contributed to the article and approved the submitted version.

## Funding

This project was funded by the Arts and Humanities Research Council (AHRC) as part of UK Research & Innovation COVID-19 funding.

## Conflict of Interest

The authors declare that the research was conducted in the absence of any commercial or financial relationships that could be construed as a potential conflict of interest.

## Publisher's Note

All claims expressed in this article are solely those of the authors and do not necessarily represent those of their affiliated organizations, or those of the publisher, the editors and the reviewers. Any product that may be evaluated in this article, or claim that may be made by its manufacturer, is not guaranteed or endorsed by the publisher.

## Footnotes

^1^Available online at: https://www.liverpoolworldheritage.com/explore-our-world-heritage-site/cultural-quarter/.

^2^Available online at: https://www.cultureliverpool.co.uk/news/culture-delivers-massive-boost-for-city/.

^3^Available online at: https://www.liferooms.org.
