# Supplementary material for: Regional Innovation in Arts Provision Spawned by COVID-19: “It Became a Lifeline for a Lot of People Who Are Stuck at Home”
Source: Front Public Health. 2022 Feb 17;10:753973. doi: 10.3389/fpubh.2022.753973 (PMC8891434; doi:10.3389/fpubh.2022.753973)
Supplement: Supplementary file 1 [file Table_1.DOCX]

**Survey 1 Interview Schedule (Wave 1)**

**Welcome and Introduction**

**Role**

What is your role at [arts/cultural organization]?

**Perceptions**

What has been the impact of COVID-19 lockdown on usual provision? Which activities or events has your organization been unable to provide as usual? [Probe for: Justification, criteria on prioritization, decision-making processes and structures]

What has been the impact of COVID-19 lockdown on access to arts provision for those who usually access the arts through formal healthcare routes?

What impact do you think restricted access to usual provision has had on people’s mental health and wellbeing?

Which populations do you feel will have been most affected by not being able to access your usual provision?

How do you analyze your audiences/reach? What data do you collect and how do you collect this data?

**Service Provision**

Can you describe any alternative measures that your organisation has put in place to try to continue service provision? [Probe for: Innovation in modes of delivery, technical challenges]

If applicable: How are you reaching people in mental health settings?

Did you involve/speak to your audiences/beneficiaries to seek their views when making these changes? / What is the involvement of exhibitors or other users of space?

What impact has this alternative form of provision had on people’s mental health and wellbeing?

How has your organisation communicated this alternative provision to its (usual or new) audiences? [Probe for: Use of different media, intermediaries, cooperation with other arts organisations, campaigns, local authorities, schools, etc]

Can you describe any successes in this process? (e.g., did any new modes of provision, or of communicating them, work particularly well in reaching former or new or hard to reach audiences?)

Can you describe any challenges in this process? If so, how did your organisation seek to overcome particularly challenging accessibility issues?

**Evaluation/Reflection**

Has this process led the organisation to reflect on its role as an arts/cultural provider?

Is there anything else you would like to say?

**Thank you and Debrief**
